# Supplementary material for: Multi‐dataset identification of innovative feature genes and molecular mechanisms in keratoconus
Source: J Cell Mol Med. 2024 Sep 19;28(18):e70079. doi: 10.1111/jcmm.70079 (PMC11412914; doi:10.1111/jcmm.70079)
Supplement: Supplementary file 2 — Material S2. [file JCMM-28-e70079-s002.zip › 7.GSEA analysis/5.RNF217-AS1/KEGG_ASCORBATE_AND_ALDARATE_METABOLISM.html]

Details for gene set KEGG\_ASCORBATE\_AND\_ALDARATE\_METABOLISM[GSEA]

|  || Dataset | RNF217-AS1.group.cls#h\_versus\_l |
| Phenotype | group.cls#h\_versus\_l |
| Upregulated in class | h |
| GeneSet | KEGG\_ASCORBATE\_AND\_ALDARATE\_METABOLISM |
| Enrichment Score (ES) | 0.27678922 |
| Normalized Enrichment Score (NES) | 0.7397492 |
| Nominal p-value | 0.83693516 |
| FDR q-value | 1.0 |
| FWER p-Value | 1.0 |
Table: GSEA Results Summary

  

Fig 1: Enrichment plot: KEGG\_ASCORBATE\_AND\_ALDARATE\_METABOLISM      
 Profile of the Running ES Score & Positions of GeneSet Members on the Rank Ordered List

  

| SYMBOL | TITLE | RANK IN GENE LIST | RANK METRIC SCORE | RUNNING ES | CORE ENRICHMENT || 1 | UGT1A8 | na | 901 | 0.455 | 0.1390 | Yes |
| 2 | UGT2B4 | na | 3115 | 0.286 | 0.1791 | Yes |
| 3 | UGT1A6 | na | 4124 | 0.248 | 0.2401 | Yes |
| 4 | UGT2B11 | na | 5963 | 0.184 | 0.2541 | Yes |
| 5 | UGT2B28 | na | 7074 | 0.151 | 0.2768 | Yes |
| 6 | UGT2B10 | na | 9434 | 0.097 | 0.2439 | No |
| 7 | UGT2A3 | na | 10034 | 0.086 | 0.2578 | No |
| 8 | UGT2A1 | na | 14187 | 0.028 | 0.1485 | No |
| 9 | UGDH | na | 18451 | -0.015 | 0.0312 | No |
| 10 | ALDH3A2 | na | 20121 | -0.031 | -0.0056 | No |
| 11 | ALDH9A1 | na | 23254 | -0.065 | -0.0723 | No |
| 12 | ALDH7A1 | na | 25259 | -0.095 | -0.0957 | No |
| 13 | UGT2B7 | na | 26756 | -0.121 | -0.0949 | No |
| 14 | UGT2B15 | na | 26916 | -0.124 | -0.0545 | No |
| 15 | ALDH1B1 | na | 28642 | -0.166 | -0.0439 | No |
| 16 | MIOX | na | 29152 | -0.180 | 0.0070 | No |
| 17 | ALDH2 | na | 33631 | -0.423 | 0.0316 | No |
Table: GSEA details [plain text format]

  

Fig 2: KEGG\_ASCORBATE\_AND\_ALDARATE\_METABOLISM      
 Blue-Pink O' Gram in the Space of the Analyzed GeneSet

  

Fig 3: KEGG\_ASCORBATE\_AND\_ALDARATE\_METABOLISM: Random ES distribution      
 Gene set null distribution of ES for **KEGG\_ASCORBATE\_AND\_ALDARATE\_METABOLISM**
